# Metabolic rate does not scale with body size or activity in some tick species

**DOI:** 10.1007/s10493-024-00958-9

**Published:** 2024-09-17

**Authors:** Kayla N. Earls, Kennan J. Oyen

**Affiliations:** 1 Department of Veterinary Microbiology and Pathology, Washington State University, Pullman, WA 99164-7040 USA; 2 Animal Diseases Research Unit, Department of Agriculture, Agricultural Research Service, 3003 ADBF, Pullman, WA 99164-6630 USA

**Keywords:** Dermacentor, Haemaphysalis, Rhipicephalus, Flow-through respirometry

## Abstract

Respiration in ticks is highly efficient and exceptionally low. Ticks can survive years between bloodmeals by having low activity and respiration to conserve energetic resources. Our objective was to compare metabolic (VCO_2_) and activity rates across 6 tick species. We predicted that VCO_2_ would be different among species and scale linearly with activity and body mass. Activity and CO_2_ production were measured for 32 h in 6 tick species: *Dermacentor andersoni*, *D. variabilis*, *Haemaphysalis longicornis*, *Rhipicephalus appendiculatus*, *R. microplus*, and *R. sanguineus*. Individual ticks were measured for 30 min three times to ensure breathing occurred. Absolute and mass-specific VCO_2_, total activity, body mass, and ventilation patterns were compared among species. As expected, ticks did not always breathe during the 30-minute measurements, especially *R. sanguineus*. Ventilation patterns differed among species with *R. microplus* having primarily cyclic patterns and *R. appendiculatus* having discontinuous gas exchange. VCO_2_ did not scale with body mass in most species. *Haemaphysalis longicornis* and *R. sanguineus* had the lowest VCO_2_; however, *H. longicornis* was the second most active species. Life history, including questing behavior and range expansion, could be contributing to differences between species. For instance, *H. longicornis* had exceptionally low metabolic rates despite above average activity levels, suggesting an energetic advantage which may underlie recently documented range expansions in North America. Our results demonstrate how ticks utilize energetic resources to maximize longevity. Future research describing questing behavior and distribution modeling may help explain differences in metabolic rates and activity and impacts on life history traits.

**Supplementary Information:**

The online version contains supplementary material available at 10.1007/s10493-024-00958-9.

## Introduction

Ticks are blood feeding ectoparasites that transmit numerous pathogens to vertebrate hosts. Ixodid ticks (hard ticks) only feed three times in their lifespan and can survive for years despite feeding infrequently (Apanaskevich and Oliver 2014). During this time, ticks need to preserve energetic resources to survive and locate a host for bloodmeals. One method of energy conservation is decreasing metabolic rate (Lighton et al. 2001). Changes in metabolic rate can have lasting effects on longevity, activity, and reproduction (Reinhold 1999). For small organisms, having lower metabolic rates can contribute to longer lifespans (Arnqvist et al. 2017; Auer et al. 2018; Pettersen et al. 2016). Ticks likely use multiple methods to conserve energy during off-host periods between bloodmeals.

Encountering a host can be unpredictable and require utilizing metabolic stores that are essential for survival; therefore, ticks have evolved physiological mechanisms to conserve valuable energy. One method of conserving energetic resources is through limited activity, which can include a sit-and-wait host-seeking strategy. This strategy is characterized by long periods of inactivity until an individual experiences a host cue, environmental cue, or when energetic reserves are reaching a critical low (Alasmari and Wall 2021; Tomkins et al. 2014). Organisms that utilize this strategy include ticks, scorpions, reptiles, and insects (Benton 1992; Kral 2013; Lighton et al. 1995; Lighton et al. 2001; Porges et al. 2003). Sit-and-wait strategists may conserve energetic resources by having lower metabolic rates and activity levels which allows for surviving long periods between feeding (Lighton and Fielden 1995). Alternatively, ticks may actively search for a host but there is a higher energetic cost associated with this behavior. The degree to which metabolic rates scale with activity among tick species is unknown but likely has direct consequences on tick host seeking behavior and longevity.

Ticks have exceptionally low metabolic rates, respiring between 0.186 and 1.334 µl/h, which is 12% lower than other small arthropods, including spiders, ants, and beetles (Lighton and Fielden 1995). Gas exchange is controlled by opening and closing of spiracle plates at the spiracle opening in adult ticks allowing for different types of ventilation patterns depending on energy demand (Fielden and Duncan 2014). To maintain such low metabolic rates ticks likely utilize discontinuous gas exchange in times of low energy demand. Infrequent breathing allows ticks to survive long bouts submerged under water (Fielden et al. 2011; Gianneli et al. 2012; Sá-Hungaro et al. 2014; Sullivan et al. 2022). For instance, 50% of measured *Dermacentor variabilis* can survive up to 11 days submerged in water (Fielden et al. 2011). Additionally, their respiratory system needs to be flexible to allow for long periods of suppressed respiration followed by periods of rapid expansion and compaction once they finally feed (Fielden and Duncan 2014; Randolph 2009). Low metabolic rates align with their extended life history with limited opportunities to feed. Low metabolic rates are not uncommon in other small haematophagous insects and arthropods. For instance, mosquitos are smaller than ticks and have even lower metabolic rates despite the energy demand for flight (Gray and Bradley 2003; Huestis et al. 2011). In comparison, kissing bugs (*Rhodnius prolixus*) have slightly larger masses than many unfed ticks and are more active through various modes of locomotion but still have lower metabolic rates (Contreras and Bradley 2009; Rolandi et al. 2014). Across haematophagous arthropods, metabolic rate drastically increases with blood feeding (Fielden et al. 1994; Gray and Bradley 2006; Landulfo et al. 2019; Leis et al. 2016).

Understanding and quantifying metabolic rates in tick species can be used to understand costs of host seeking behavior and longevity. We hypothesized that metabolic rate would scale with body size with larger ticks having higher VCO_2_ (volume of CO_2_ used to determine metabolic rate). Additionally, VCO_2_ should scale positively with activity. Our objective was to quantify metabolic rates and characterize ventilation and activity patterns in six tick species in the genera *Dermacentor*, *Haemaphysalis*, and *Rhipicephalus*. To determine differences in metabolic rate between species, CO_2_ emissions (VCO_2_) were measured along with ventilation patterns, body mass, and activity.

## Methods

### Ticks

Adult *Rhipicephalus microplus*, *Rhipicephalus appendiculatus*, *Dermacentor andersoni*, and *Haemaphysalis longicornis* were arbitrarily selected from colonies maintained at the Holm Research Center at the University of Idaho in Moscow, ID, USA. Ticks were stored in an incubator at 26 °C with 12:12 L: D. Ticks of each species were previously fed on a bovine calf as nymphs and molted to adults. The time since feeding and molting to adulthood (referred to as age) varied between each species (*D. andersoni* 181 days, *H. longicornis* 440 days, *R. appendiculatus* 597 days, and *R. microplus* 0 days). *Rhipicephalus microplus* does not survive long off host; therefore, *R. microplus* adults were measured immediately after molting to adulthood and before re-attaching to the host.

Adult *D. variabilis* and *R. sanguineus* ticks were purchased from the Tick Rearing Facility at Oklahoma State University (Stillwater, OK, USA). All ticks were weighed to the nearest 0.01 mg before and after the experiment using an analytical balance (Sartorius QUINTIX224-1 S, Göttingen, Germany).

### Flow-through respirometry

Sixteen ticks, alternating males (*n* = 8) and females (*n* = 8) of the same species, were individually placed into 2.5 ml glass tubes that were sealed with rubber stoppers and connected to the MAVEn (Sable Systems, Las Vegas, NV, USA). Fine mesh on the stoppers prevented ticks from escaping. Only female *H. longicornis* were measured because males are rare due to their parthenogenetic life history (Chen et al. 2012). Ticks were allowed to acclimate to their chamber and air flow for 30 min before measurements began.

A pump (SS4 Subsampler, Sable Systems) pulled room air from a ventilation duct through two columns of drierite and ascarite to scrub out water and CO_2_ before funneling air into the MAVEn. The pump ran at 500 ml/min at 30% with the flow rate being dividing amongst 16 chambers so that each tick received a continuous 5.0 ± 0.1 ml/min of oxygen. The baseline was measured between each chamber reading for 10 min to stabilize CO_2_ readings. Tick chambers were measured every second for 30 min. Each species was measured for 32 h with individual chambers being measured three times throughout the test period. Temperature (°C), light (lux), humidity (percent), and air pressure (kPa) were continuously measured every second during trials. All trials were conducted at room temperature (21.5 ± 0.14 °C) with constant light (9.94 ± 0.06 lx). Average room humidity was 17.9 ± 1.80% while barometric pressure 92.9 ± 0.16 kPa.

The MAVEn was connected via analog to a CO_2_ analyzer (LiCor 850, LI-COR Biosciences, Lincoln, NE, USA) and zeroed before trials to quantify CO_2_ emissions. CO_2_ in parts per million was recorded by the MAVEn software and exported to EXPEDATA (PRO Version 1.9.27, Sable Systems). Due to the low flow rate creating lags in CO_2_ readings, the first 5 min of each animal and baseline chamber were removed prior to being uploaded to EXPEDATA. The rate of CO_2_ emissions (VCO_2_ ml/hr) was calculated by using the following formula:$\eqalign{\>\mathop {\rm{V}}\limits^. {\rm{C}}{{\rm{O}}_2}\>\left( {{{ml} \over {hr}}} \right) & = \left( {C{O_2}\left( {ppm} \right) \div {\rm{1,000,000}} \times \>chamber\>flow\>rate\>\left( {{{ml} \over {min}}} \right) \times \>60\>mins} \right) \cr & \quad - baseline\left( {{{ml} \over {hr}}} \right) \cr}$

**Fig. 1:**
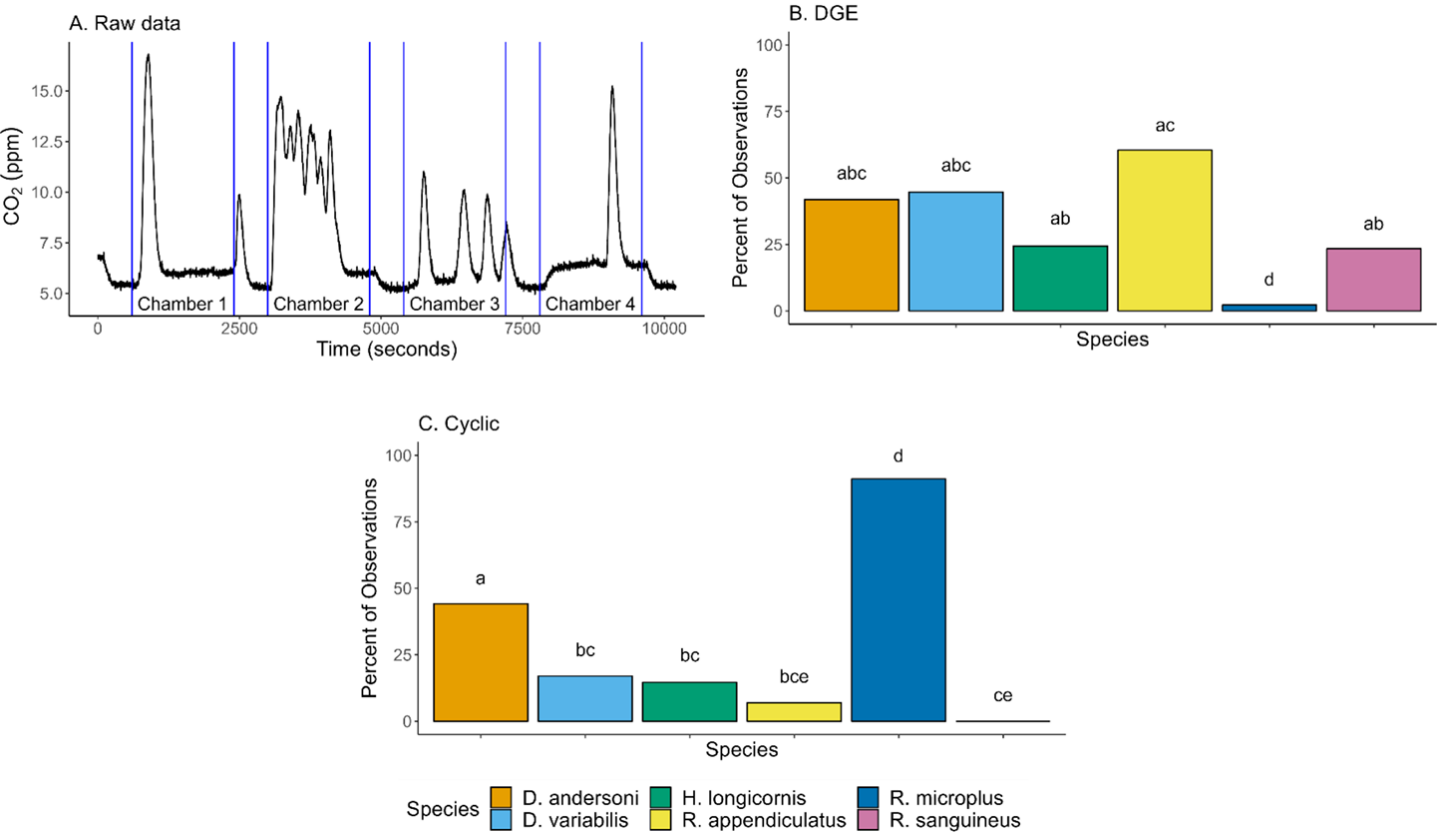
(**A**) Raw CO_2_ output example from *D. variabilis*. Blue vertical lines indicate the start and stop of baseline and chamber readings. The percent of 30-minute chamber readings where ticks (*n* = 16 ticks per each species, except for *R. microplusn* = 32) had either discontinuous gas exchange (**B**) or cyclic (**C**) ventilation patterns. Boxes with different letters are significantly different from each other (*p* < 0.05)

Mass-specific metabolic rate per individual (ml/hr/g) was calculated by dividing $\:\dot{\text{V}}$VCO_2_ by the tick’s average mass (grams). Average mass was calculated by using individual tick mass before and after VCO_2_ and activity readings. Baseline was determined by averaging readings from the baseline chamber before and after each animal chamber. Ventilation type was determined visually by whether there was a single peak (discontinuous gas exchange (DGE)) or cyclic pattern (Fig. 1A).

### Activity

Activity was continuously measured for all chambers regardless of which chamber was being read for CO_2_ emission. Under each chamber were 3 infrared sensors that measured activity every second. Activity was measured by whether a tick obstructed the infrared beam. Average activity was calculated during the 30 min intervals the ticks were measured. Total activity was calculated by taking the sum of activity readings over the entire 32-hour trial.

### Statistical analysis

All means are presented as ± standard error of the mean (S.E.M.), and significance was determined by *p* < 0.05. All statistical analyses were performed in R (Version 4.2.2; R Core Team, 2022) using the following packages: *ggplot2*, *ggpubr*, *dplyr*, *nlme*, *multcomp*, *emmeans*, and *rcompanion*. An ANOVA was performed to compare wet body mass across species and sex. A t-test was then used to determine overall sex differences and within each species. A binomial logistic regression was used to determine the likelihood of ticks having either DGE or cyclic ventilation patterns during each 30-minute measurement. Ventilation pattern data was then presented as a percent to show how many 30-minute observations had either a DGE or cyclic pattern.

Mass, VCO_2_, and total activity were log transformed for the remainder of the analyses. Linear mixed models were performed to determine the significant factors influencing absolute VCO_2_ and mass-specific VCO_2_. Individual ticks were measured 3 times during the 32 h they were in the MAVEn, which was included as a random effect in the models to account for repeated measures. All nonsignificant factors were removed from the final models. Total activity across species was tested using an ANOVA. A mixed model was not used because total activity was calculated by the summation of activity over the entire 32 h the ticks were in the MAVEn; therefore, replicate was not needed as a random factor. Tukey post-hoc comparisons were used to detect significant differences between species. To determine if differences in age was impacting VCO_2_ and activity, ANOVA was used to compare among species. Linear regression revealed effects of age on VCO_2_ and mass-specific VCO_2_ and activity varied significantly among species, so residuals from the regression of response variables on age were compared among species by ANOVA followed by Tukey HSD post-hoc tests. Using residuals essentially corrects data for age, allowing comparisons among species without the impact of age.

## Results

### Wet body mass

Mass significantly differed between species (ANOVA, F_5,85_ = 56.5, *p* < 0.0001; Supplemental Fig. 1). Overall, *D. variabilis* were the largest ticks (6.11 ± 0.356 mg) followed by *D. andersoni* (4.14 ± 0.287 mg). *Rhipicephalus appendiculatus* (3.88 ± 0.288 mg) and *R. sanguineus* (3.12 ± 0.185 mg) were in the middle range of measured body sizes, while *H. longicornis* (1.96 ± 0.028 mg) and *R. microplus* (1.53 ± 0.131 mg) were the smallest.

Body mass differed between sexes in some species, but not others. Female ticks were larger in *D. andersoni* (females = 4.96 ± 0.192 mg, males = 3.21 ± 0.293 mg; t = 4.99, df = 10.6, *p* = 0.0005) and *R. microplus* (females = 1.86 ± 0.008 mg, males = 1.15 ± 0.231 mg; t = 2.90, df = 16.0, *p* = 0.010). Contrarily, males were larger than females in *R. appendiculatus* (females = 3.09 ± 0.009 mg, males = 4.79 ± 0.334 mg; t = -4.88, df = 5.84, *p* = 0.003) and *R. sanguineus* (females = 2.92 ± 0.184 mg, males = 3.70 ± 0 mg; t = -4.22, df = 5, *p* = 0.008). There were no significant differences in mass between male and female *D. variabilis* (females = 6.6 ± 0.639 mg, males = 6.06 ± 0.414 mg; t = 0.134, df = 10.5, *p* = 0.896) and only female *H. longicornis* (1.96 ± 0.028 mg) were measured.

### Ventilation patterns

Each 30-minute measurement was separated into three categories: no ventilation patterns, discontinuous gas exchange, or cyclic. More than one type during the 30-minute window was not observed. Each 30-minute chamber reading was then characterized as an observation. Ticks have low metabolic rates and often did not have detectible ventilation patterns; therefore, the addition of DGE and cyclic percentages may not equal 100%. DGE or cyclic ventilation patterns did not vary between sexes in any species.

Likelihood of having DGE ventilation patterns during a 30-minute measurement was significantly different across species (*X*^2^ = 63.07, df = 5, *p* < 0.0001; Fig. 1B). Post hoc analysis determined there were no significant differences between the *Dermacentor* species. *Dermacentor andersoni* had DGE patterns 41.9% of the total observations, while *D. variabilis* experienced DGE patterns 44.7% of observations (Fig. 1B). *Rhipicephalus* species had differences in DGE ventilation patterns across the 3 species (Fig. 1B). *Haemaphysalis longicornis* had 24.4% of observation being DGE patterns. *Rhipicephalus appendiculatus* (60.5% of observations) were 30 times more likely to have DGE patterns compared to *R. microplus* (2.22%) and 2.6 times more likely than *R. sanguineus* (23.4%). *Rhipicephalus sanguineus* was the only species to have only one type of ventilation pattern (DGE). Overall, *R. appendiculatus* had the most observations, while *R. microplus* had the least.

Likelihood of having cyclic ventilation patterns during a 30-minute measurement was also significantly different across species (*X*^2^ = 173.2, df = 5, *p* < 0.0001; Fig. 1C). *Dermacentor andersoni* (44.2% of observations) and *R. microplus* (91.1%) were more likely to have cyclic patterns compared to the other measured species (Fig. 1C). Only *D. andersoni* (41.9% DGE, 44.2% cyclic) and *R. microplus* (2.22% DGE, 91.1% cyclic) had more cyclic pattern observations compared to DGE. The percentage of cyclic pattern observations remained low for *D. variabilis* (17.0%), *H. longicornis* (14.6%), *R. appendiculatus* (6.98%), and *R. sanguineus* (0.00%; Fig. 1C). Cyclic ventilation patterns were evenly observed across replicates, except for *D. andersoni* which were less likely to have the pattern in the third replicate. The length of the experiment may contribute to differences in replicate 1 and 3.

Overall, some species were more likely to have one ventilation pattern compared to the other and breathing was not always detected during the 30-minute observation. Most of the tick species had more observations of DGE ventilation patterns than cyclic, except for *D. andersoni* and *R. microplus* (Fig. 1B and C). *Rhipicephalus sanguineus* was the only species to have only one type of ventilation pattern (DGE) and had the least number of breathing observations (Fig. 1B and C). *Rhipicephalus microplus* had the highest percentage of cyclic observation.

### Absolute VCO2

**Fig. 2:**
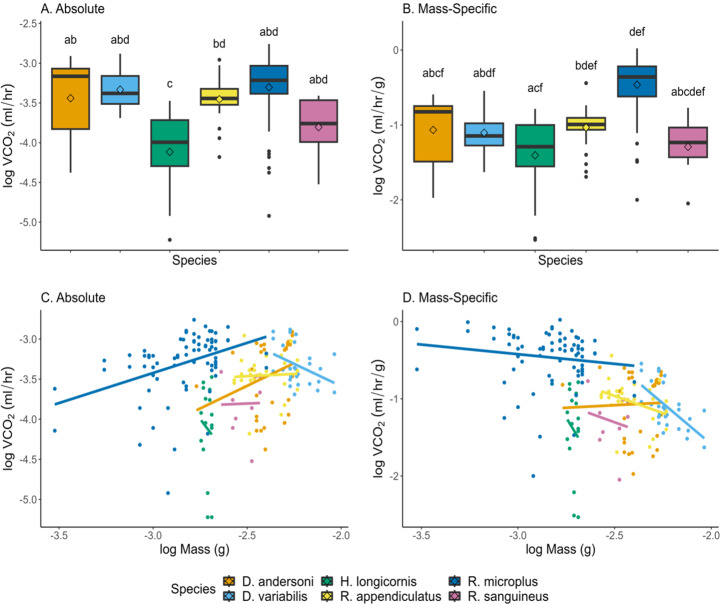
Comparison of absolute and mass-specific VCO_2_ across species. (**A**) Absolute VO_2_ across measured *Dermacentor sp*., *H. longicornis*, *and Rhipicephalus sp*. (**B**) Mass-specific VO_2_ across tick species. Boxes represent 1st to 3rd quartile with the median indicated by the center line and mean by the diamonds. Extending lines represent 25th to 75th percentiles of the data. Points beyond are considered outliers. Boxes with different letters are significantly different from each other (*p* < 0.05). (**C**) Scaling of absolute VO_2_ with mass by each tick species. Only *D. variabilis* and *R. microplus* have significant regressions (Table 2). (**D**) Scaling of mass-specific VO_2_ with mass by each tick species. Only *D. variabilis* has a significant regression (Table 2)

Absolute metabolic rate varied by species, mass, and ventilation type (Fig. 2A; Table 1). Excluding *H. longicornis*, females (0.648 ± 0.040 µl/hr) had higher VCO_2_ than males (0.420 ± 0.028 µl/hr; t = 3.25, df = 161, *p* = 0.001; Supplemental Fig. 2). Using a t test to investigate sex differences within each species, only *R. microplus* has significant sex differences with females having higher absolute VCO_2_ than males (0.812 ± 0.055 µl/hr; 0.422 ± 0.039 µl/hr respectively; t = 4.21, df = 45.7, *p* = 0.0001). Overall, *D. variabilis* (0.550 ± 0.066 µl/hr), *R. microplus* (0.646 ± 0.043 µl/hr), and *D. andersoni* (0.536 ± 0.066 µl/hr) had the highest metabolic rates (Fig. 2A). Absolute VCO_2_ was not significantly different between *Dermacentor* species and *R. microplus* despite *R. microplus* almost exclusively respiring cyclically. *Rhipicephalus sanguineus* (0.201 ± 0.043 µl/hr), *H. longicornis* (0.129 ± 0.026 µl/hr), *R. appendiculatus* (0.405 ± 0.042 µl/hr) had the lowest metabolic rates (Fig. 2A). *Rhipicephalus sanguineus* was not significantly different from the other species likely due to the low sample size. *Rhipicephalus microplus* was expected to have a higher VCO_2_ compared to the other *Rhipicephalus* species because the ticks were measured shortly after feeding.

**Table 1:** Global models for absolute and mass-specific VCO_2_

	Model Term	Estimate	Std error	T value	*P* value
Absolute VCO_2_	Intercept	-2.03	0.370	-5.48	< 0.0001
	Species (*D. variabilis*)	0.170	0.085	1.99	0.0478
	Species (*H. longicornis*)	-0.344	0.108	-3.19	0.0017
	Species (*R. appendiculatus*)	0.286	0.086	3.31	0.0011
	Species (*R. microplus*)	0.267	0.099	2.70	0.0075
	Species (*R. sanguineus*)	-0.054	0.125	0.433	0.6652
	Log mass (g)	0.735	0.153	4.81	< 0.0001
	Cyclic ventilation	0.550	0.066	8.31	< 0.0001
Mass-Specific VCO_2_	Intercept	-1.40	0.084	-16.7	< 0.0001
	Species (*D. variabilis*)	0.132	0.083	1.59	0.1130
	Species (*H. longicornis*)	-0.254	0.095	-2.67	0.0083
	Species (*R. appendiculatus*)	0.299	0.086	3.46	0.0007
	Species (*R. microplus*)	0.390	0.070	5.58	< 0.0001
	Species (*R. sanguineus*)	0.092	0.123	0.74	0.4592
	Cyclic ventilation	0.553	0.067	8.31	< 0.0001

Body mass was not a significant factor determining differences in VCO_2_ when comparing species. When investigating species individually, only *D. variabilis* (F_1,26_ = 4.56, *p* = 0.04) and *R. microplus* (F_1,74_ = 13.7, *p* < 0.001) had a linear relationship with VCO_2_ and body mass (Fig. 2C; Table 2). Interestingly, *R. microplus* had a positive relationship suggesting that as body size increases, VCO_2_ also increases (Fig. 2C; Table 2). Interestingly, *D. variabilis* had a negative relationship with larger ticks having lower metabolic rates. VCO_2_ did not scale with the other four species, which was unexpected (Fig. 2C; Table 2). The relationship may not be present due to small variation in body size and VCO_2_.

**Table 2:** Linear regressions for absolute and mass-specific VCO_2_ across species (Fig. 2C, D). Reported F and p values are shown for each linear equation. Post hoc letters indicate significant differences between species in the overall ANCOVA model comparing regressions of species

VCO_2_	Species	Equation (y = mx + b)	AIC	*R* ^2^	F value	Post hoc
Absolute	*D. andersoni*	1.13x– 0.75	41.8	0.05	F_1,30_ = 2.63	ab
	*D. variabilis*	-1.12x– 5.81	1.84	0.12	F_1,26_ = 4.64*	abd
	*H. longicornis*	-3.14x– 12.6	31.8	0.06	F_1,14_ = 0.20	c
	*R. appendiculatus*	0.11x– 1.18	6.40	0.04	F_1,26_ = 0.06	ab
	*R. microplus*	0.75x– 1.17	65.0	0.14	F_1,74_ = 13.5***	bd
	*R. sanguineus*	0.10x– 3.56	12.2	0.14	F_1,7_ = 0.003	c
Mass-Specific	*D. andersoni*	0.13x– 0.75	41.8	0.03	F_1,30_ = 0.04	abd
	*D. variabilis*	-2.11x– 5.81	1.83	0.36	F_1,26_ = 16.4***	abe
	*H. longicornis*	-4.14x– 12.6	31.8	0.05	F_1,14_ = 0.35	cf
	*R. appendiculatus*	-0.89x– 3.18	6.40	0.10	F_1,26_ = 3.99	abd
	*R. microplus*	-0.25x– 1.17	64.5	0.01	F_1,74_ = 1.52	bef
	*R. sanguineus*	-0.90x– 3.56	12.2	0.10	F_1,7_ = 0.25	cf

* *p* < 0.05, ** *p* < 0.01, *** *p* < 0.001

### Mass-specific VCO2

Species and ventilation type were the only significant factors in determining mass-specific metabolic rate (Fig. 2B; Table 1). There were no overall sex differences in mass-specific VCO_2_ between males and females, even when excluding *H. longicornis* (t = 0.220, df = 169, *p* = 0.83). *Haemaphysalis longicornis* and *R. sanguineus* had the lowest mass-specific metabolic rates (66.6 ± 13.3 µl/g/hr; 66.8 ± 16.1 µl/g/hr respectively) compared to all the other species. There were no significant differences between the *Dermacentor* species (*D. andersoni* 122.6 ± 14.2 µl/g/hr; *D. variabilis* 99.3 ± 14.1 µl/g/hr), however, there were differences between *Rhipicephalus* species. Results from the linear mixed model show that *R. sanguineus* (66.8 ± 16.1 µl/g/hr) had significantly lower mass-specific metabolic rate compared to *R. appendiculatus* (108.7 ± 12.1 µl/g/hr) and *R. microplus* (435.1 ± 27.2 µl/g/hr). *Rhipicephalus appendiculatus* and *R. microplus* were not different from each other when the linear mixed-effect model included ventilation patterns.

VCO_2_ varied linearly with body mass between species (F_5,178_ = 24.9, *p* < 0.0001). Sex, ventilation type, and replicate influences species differences. ANCOVA was ran without *H. longicornis* and sex remained significant, so the species was left in the analysis. Differences between replicate 1 and 3 contributed to significance. Only *D. variabilis* had a significant linear relationship with mass-specific VCO_2_ and mass (Fig. 2D; Table 2). Similarly, *D. variabilis* has a negative relationship with larger ticks having lower mass-specific VCO_2_ than smaller ticks.

### Activity

**Fig. 3:**
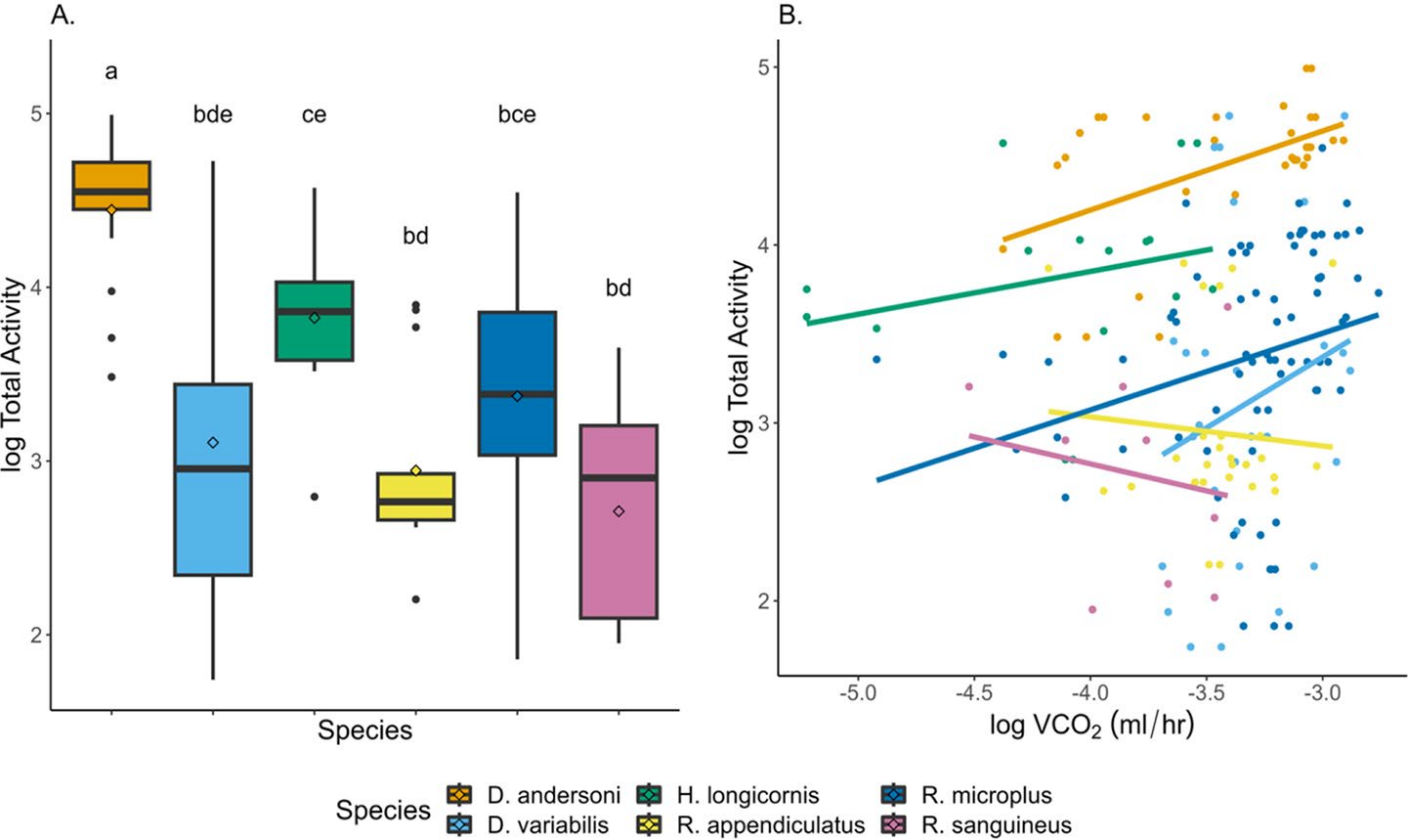
Differences in total activity based on species. (**A**) Total activity differed between tick species. Boxes with different letters are significantly different from each other (*p* < 0.05). Means are indicated by diamonds. (**B**) Total activity scaled linearly with VCO_2_ in 2 tick species (*D. andersoni* and *R. microplus*). Linear regression equations with statistics and post hoc analysis can be found in Table 3

Mean activity during the 30-minute observations was low across tick species. Because ticks are generally inactive, we used total activity across the entire experiment (32 h) to estimate differences among species. Total activity per individual over the course of the 32-hour measurements was significantly different among species (ANOVA; F_5,84_ = 15.0, *p* < 0.0001; Fig. 3A). *Dermacentor andersoni* was the most active (4.68e^4^ ± 8.47e^3^ arbitrary units) followed by *H. longicornis* (1.17e^4^ ± 4.44e^3^), *D. variabilis* (1.01e^4^ ± 7.83e^3^), *R. microplus* (6.84e^3^ ± 1.35e^3^), *R. appendiculatus* (2.79e^3^ ± 1.08e^3^), and *R. sanguineus* (1.37e^3^ ± 759; Table 3; Fig. 3C). Overall, the *Dermacentor sp.* and *H. longicornis* had the highest total activity across tick species. Differences in activity among species was partly driven by sex (ANOVA; F_1,84_ = 11.5, *p* = 0.001; Supplemental Fig. 2). Interestingly, females were more active than males in all three *Rhipicephalus* species, but not *Dermacentor*. *Haemaphysalis longicornis* was excluded due to only females being measured. Sex difference accounts for the large variation in *R. microplus* (female activity: 7280 ± 931; male activity: 1348 ± 254; t = 7.79, df = 47.7, *p* < 0.0001), but not *D. variabilis* (female activity: 9210 ± 5417; male activity: 7703 ± 3276; t = 0.12, df = 30.0, *p* = 0.91). *Dermacentor variabilis* and *R. microplus* had large variations in total activity with some individuals being highly active throughout the test period and others relatively immobile.

**Table 3:** Linear regression for changes in log VCO_2_ with total activity for each of the measured tick species. Post hoc letters indicate significant differences between species in the overall ANCOVA model comparing regressions of species

Species	Equation (y = mx + b)	AIC	*R* ^2^	F value	Post hoc
*D. andersoni*	0.44x + 5.97	27.6	0.23	F_1,30_ = 10.0**	abd
*D. variabilis*	0.80x + 5.76	78.1	0.01	F_1,26_ = 1.27	ab
*H. longicornis*	0.24x + 4.81	29.0	0.00	F_1,14_ = 0.98	cd
*R. appendiculatus*	-0.16x + 2.38	46.3	0.03	F_1,26_ = 0.169	abd
*R. microplus*	0.43x + 4.80	147	0.06	F_1,74_ = 5.53*	ab
*R. sanguineus*	-0.30x + 1.56	21.2	0.11	F_1,7_ = 0.24	acd

* *p* < 0.05, ** *p* < 0.01, *** *p* < 0.001

Overall, metabolic rate did not scale with activity (F_1,180_ = 6.35, *p* = 0.01) which may be due to differences among species and ventilation types. Total activity scaled linearly with absolute VCO_2_ in *D. andersoni* and *R. microplus*. Slopes were significantly different between species (ANCOVA; F_5,180_ = 21.6, *p* < 0.0001; Fig. 3B). Across all species, VCO_2_ scaled linearly with activity when ticks were respiring cyclically (F_1,109_ = 5.93, *p* = 0.02) with ticks having higher VCO_2_ when they are more active. A negative linear relationship was seen between activity and VCO_2_ during discontinuous gas exchange (F_1,76_ = 8.54, *p* = 0.005).

### Age

**Fig. 4:**
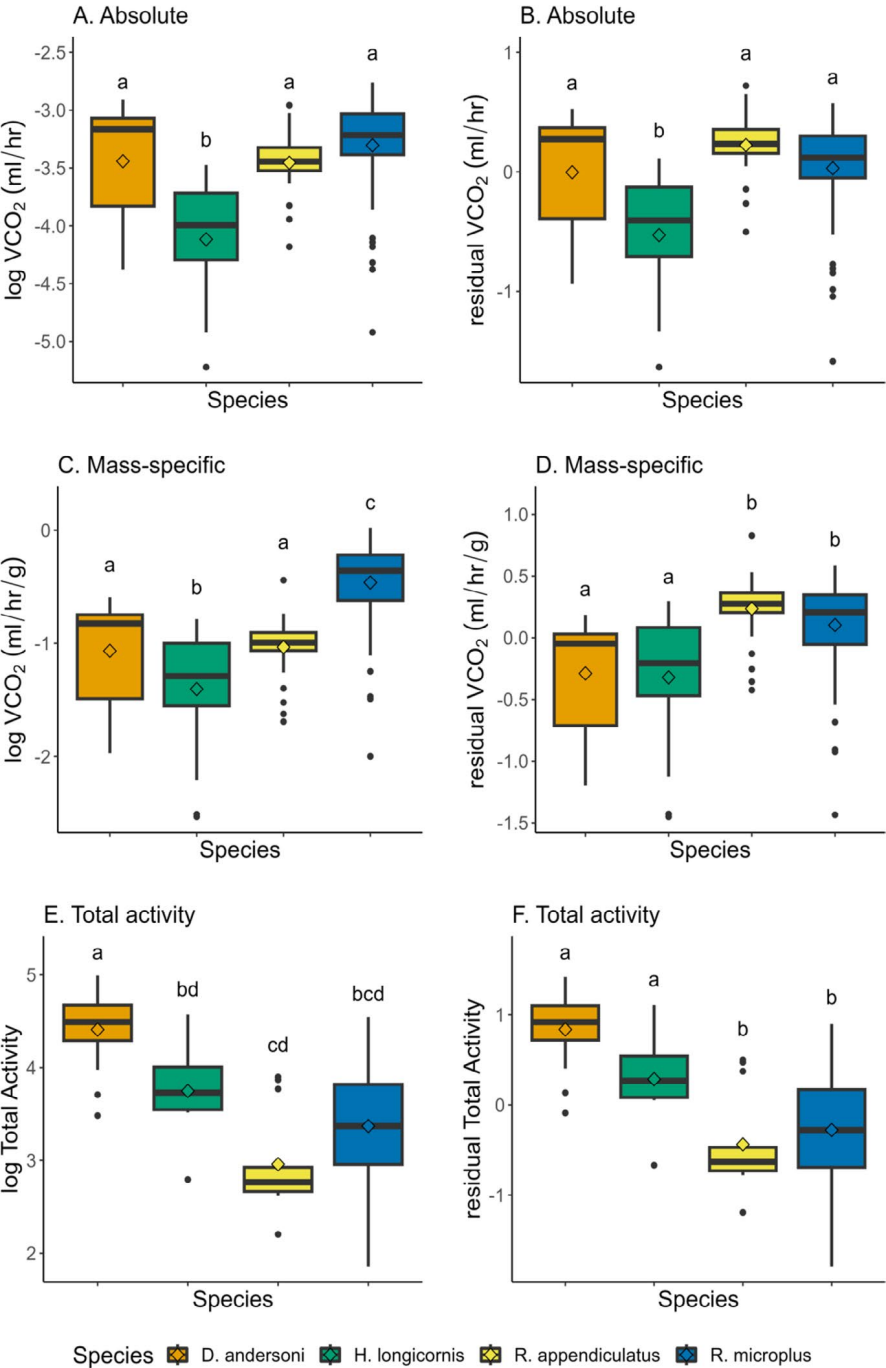
Differences in VCO_2_ and activity for log-transformed data (column 1) and log-transformed data corrected for age (column 2). (**A**) and (**B**) show absolute VCO_2_ across tick species with known times since last feeding. (**C**) and (**D**) show mass-specific VCO_2_ and total activity in (**E** and **F**). Boxes with different letters are significantly different from each other (*p* < 0.05). Means are indicated by diamonds. Linear mixed model results for A-D can be found in Supplementary Table 1

Four of the tick species were reared in the lab with known ages (time since feeding in the nymphal stage and molting to adulthood). Time since feeding had a significant contribution to log transformed absolute (ANOVA; F_1,150_ = 13.9, *p* = 0.0003) and mass-specific VCO_2_ (ANOVA; F_1,150_ = 60.3, *p* < 0.0001). However, when correcting for time since feeding, differences among species did not change (Fig. 4A and B). Only *H. longicornis* was significantly different from the other three species (Linear mixed model, Supplemental Table 1). These results suggest that age explains some variability in VCO_2_ but does not change the observed differences among species. For mass-specific VCO_2_, correcting for age shifted *H. longicornis* and *R. appendiculatus* (the oldest species we measured) up creating two groups: (1) *D. andersoni* and *H. longicornis* and (2) *R. appendiculatus* and *R. microplus*. *Rhipicephalus appendiculatus* and *R. microplus* have higher mass-specific VCO_2_ compared to D. andersoni and H. longicornis (Fig. 4D; Linear mixed model, Supplemental Table 1). Age did not significantly contribute to variation in total activity across species (ANOVA; F_1,150_ = 2.66, *p* = 0.105). When comparing total activity corrected for age, *D. andersoni* and *H. longicornis* had significantly higher activity levels than *R. appendiculatus* and *R. microplus* (Fig. 4F; ANOVA; F_3,64_ = 17.3, *p* < 0.0001). *Rhipicephalus appendiculatus* and *R. microplus* had similar activity levels and mass-specific VCO_2_ despite being there being 597 days between when both species had last fed.

## Discussion

Ticks blood-feed intermittently and can survive long periods without feeding. Energy conservation and low metabolic rates are likely essential for surviving long periods of starvation between infrequent bloodmeals. After 36 weeks of starvation, *D. variabilis* does not have decreased survival and metabolite levels (lipids, protein, and glycogen) remain consistent (Rosendale et al. 2019). Additionally, *D. variabilis* becomes more active over time as starvation is prolonged and has increased metabolic rates (Rosendale et al. 2019). Interestingly, for the species we measured, metabolic rate decreased with age (older ticks tended to have lower metabolic rates), which matches other studies showing that older animals generally have lower metabolic rates (Gray and Bradley 2003; Hack 1997; Piiroinen et al. 2010). Regardless, it would be interesting to test the same ticks repeatedly over time to determine if metabolic rate increases with age within a species, as shown in Rosendale et al. 2019. In this study, we did not test the impact of aging on metabolic rates within the same ticks, rather we show that metabolic rates vary among species regardless of age. Age contributes to variation in VCO_2_; however, other factors, such as mass and species, may be having stronger effects. For instance, *R. microplus* and *R. appendiculatus* have similar mass-specific metabolic rates and activity despite a large age gap (Fig. 4D and F) and differences in mass (Supplemental Fig. 1).

The metabolic rate (VCO_2_) of six unfed tick species were compared for this study. Overall, *D. andersoni*, *D. variabilis*, and *R. microplus* had the highest absolute metabolic rates (Fig. 2A). Absolute metabolic rates for *D. andersoni*, *D. variabilis*, and *R. sanguineus* are similar to already published data using field and lab collected ticks (Landulfo et al. 2019; Lighton and Fielden 1995; Rosendale et al. 2019). These similarities and differences highlight the need for further comparisons of metabolic rates across ticks. *Haemaphysalis longicornis* and *R. sanguineus* had the lowest metabolic rates even when factoring in mass (Fig. 2). Absolute VCO_2_ and body mass scaled in only *D. variabilis* and *R. microplus* but had different responses (Fig. 2C). *Dermacentor variabilis* had a negative relationship with larger ticks having lower VCO_2_ compared to *R. microplus* ticks that had a positive relationship. All species, except for *D. andersoni* had a negative linear relationship with body mass and mass-specific VCO_2_, however, only *D. variabilis* had a significant relationship between VCO_2_ and mass (Fig. 2D). A negative linear slope suggests that larger ticks are metabolically more efficient per gram of tissue than smaller ticks (Niven and Scharlemann 2005). Respiratory efficiency can also be seen in underwater survival as unfed *D. variabilis* adults can survive 11 days, submerged before 50% mortality (Fielden et al. 2011). Even engorged *Amblyomma auricularium*, *R. sanguineus* and *Dermacentor albipictus* females can survive a few days submerged (Gianneli et al. 2012; Sá-Hungaro et al. 2014; Sullivan et al. 2022), suggesting that even during digestion and egg production ticks are capable of dramatically reducing respiration rates. Differences in metabolic rate between species can be attributed to differences in life history, ventilation patterns, and activity.

Discontinuous gas exchange was the most common ventilation pattern amongst species, except for *R. microplus* (Fig. 1B). Discontinuous gas exchange has been previously found to be a common ventilation pattern among ticks (Fielden and Lighton 1996; Lighton 1994; Lighton and Fielden 1993, 1995). We observed that VCO_2_ was greater during periods of cyclic gas exchange, compared with DGE, suggesting that ticks may use DGE to conserve energetic resources during periods of starvation between bloodmeals. *Dermacentor variabilis* and *R. appendiculatus* had both similar ventilation patterns and metabolic rate (both absolute and mass-specific) even though *D. variabilis* is larger than *R. appendiculatus*. Previous studies have shown that feeding increases continuous CO_2_ release in *D. variabilis* and *R. sanguineus* (Fielden et al. 1999; Landulfo et al. 2019). Given that *R. microplus* in our experiment had recently fed, it is unsurprising that we primarily observed cyclic gas exchange for this tick species (Fig. 1C).

Ventilation patterns were not always observed during the 30-minute measurements. For instance, less than 25% of *R. sanguineus* observations had discontinuous ventilation and no measurement period involving *R. sanguineus* had a cyclic pattern, with 75% of the periods having no discernable breathing. Having few observations for *R. sanguineus* resulted in low sample size and statistical power to detect differences between *R. sanguineus* and other species. Additionally, less than 50% of *H. longicornis* (40%) breathed during the observation window with discontinuous and cyclic added together. Thirty-minute intervals were the maximum amount of time allotted by the respirometer to measure a single chamber. To increase the chances of capturing ticks respiring, ticks were measured for 30 min multiple times. Lighton et al. 1993 has shown that another tick species (*Amblyomma marmoreum)* can go hours without having discontinuous gas exchange ventilation. The low number of respiration observations in *R. sanguineus* and *H. longicornis* highlights the difficulties and challenges in measuring tick respiration.

In addition to respirometry measurements, both total activity during the experiment and the mean activity during the 30-minute measurements of VCO_2_ were calculated for each individual tick. Unfed ticks are not as active when not in the presence of host stimuli. Ticks in this study were inactive and did not always respire during the 30-minute measurements; therefore, results focused on the total activity of each tick. Total activity differed between species with *D. andersoni* and *H. longicornis* being the most active during the 32-hour experiment (Fig. 3). *Rhipicephalus sanguineus* was one of the least active and had the fewest breathing episodes (Figs. 1B and C and 3A). While body size influenced metabolic rates, it did not affect total activity. For *D. andersoni* and *R. microplus*, absolute VCO_2_ scaled linearly with total activity with ticks respiring more as activity increased. Interestingly, there was no relationship between activity and metabolic rate in *D. variabilis*, *H. longicornis*, *R. appendiculatus*, and *R. sanguineus*. These results highlight how well ticks can conserve energetic resources and maintain long periods of inactivity. The relationship between activity and VCO_2_ may become more evident in future experiments where ticks are given a host cue to stimulate activity.

Differences in life history and questing behavior may be contributing to the differences in VCO_2_ across species. Using the limited published data describing questing behavior, each measured species, except for *R. microplus*, was categorized as either a sit-and-wait or hunting strategist. Sit-and-wait strategists had higher absolute VCO_2_ compared to hunting strategists (Supplemental Fig. 3). Future behavioral research needs to be done to appropriately describe and categorize questing behavior to determine if this life history trait contributes to differences in metabolic rate across species.

Determining the relationships between metabolic rates and activity may clarify how ticks will physiologically and behaviorally respond to environmental changes and potential new hosts under range expansion. As ticks move with hosts and are introduced by humans, their metabolic needs will have to adjust to new areas, environmental conditions, and potential hosts. Most of the measured species are experiencing range expansion in response to increased temperatures and the introduction to new areas and hosts by humans. For instance, human introduction of *H. longicornis* has accelerated range expansion in the United States (Rochlin 2019; Saleh et al. 2021). *Rhipicephalus sanguineus* and *D. andersoni* are experiencing northern expansion with increased temperatures (Alkishe and Peterson 2022; Dantas-Torres 2010; Pascoe et al. 2022). *Dermacentor variabilis* is also expanding northward, but also contracting at more southern ranges (Dergousoff et al. 2013; Minigan et al. 2018; Saleh et al. 2021). While *R. microplus* is experiencing range expansion in Africa, environmental conditions remain limiting factors (Estrada-Peña et al. 2006; Nyangiwe et al. 2017; Sunirai et al. 2018). Not all species are experiencing expansion. The range of *R. appendiculatus* is contracting (Nemaungwe et al. 2023; Olwoch et al. 2007). In this study, *H. longicornis* and *R. sanguineus* have similar VCO_2_ profiles; however, they are using different respiration methods. Both *H. longicornis* and *R. sanguineus* had similar number of observed DGE patterns (Fig. 1B), but *H. longicornis* also utilized cyclic respirations while *R. sanguineus* did not (Fig. 2C). Despite having similar metabolic rates, *H. longicornis* was significantly more active than *R. sanguineus*. The ability to maintain a low metabolic rate while being more active might give *H. longicornis* a physiological advantage, allowing for host seeking without depleting energetic resources and explain their recently documented rapid invasion throughout the eastern part of North America (Raghavan et al. 2019). Understanding how ticks utilize their energetic resources through measuring metabolic rates and activity and the impact of environmental stressors such as temperature and infection may facilitate accurate predictions of how climate change and anthropogenic land use will impact ticks and the pathogens they transmit in the future. Many abiotic and biotic factors can influence metabolic rate; therefore, more comprehensive studies should investigate the effects of ecological factors, such as temperature and relative humidity and physiological factors, such as infection, blood feeding, and development.

## Electronic supplementary material

Below is the link to the electronic supplementary material.

Supplementary Material 1

Supplementary Material 2

## Data Availability

The full dataset will be available from Dryad digital repository following peer review.
