# Supplementary material for: Metabolic rate does not scale with body size or activity in some tick species
Source: Exp Appl Acarol. 2024 Sep 17;93(4):869–85. doi: 10.1007/s10493-024-00958-9 (PMC11534985; doi:10.1007/s10493-024-00958-9)
Supplement: Supplementary file 1 — Supplementary Material 1 [file 10493_2024_958_MOESM1_ESM.docx]

**Supplemental Figures**


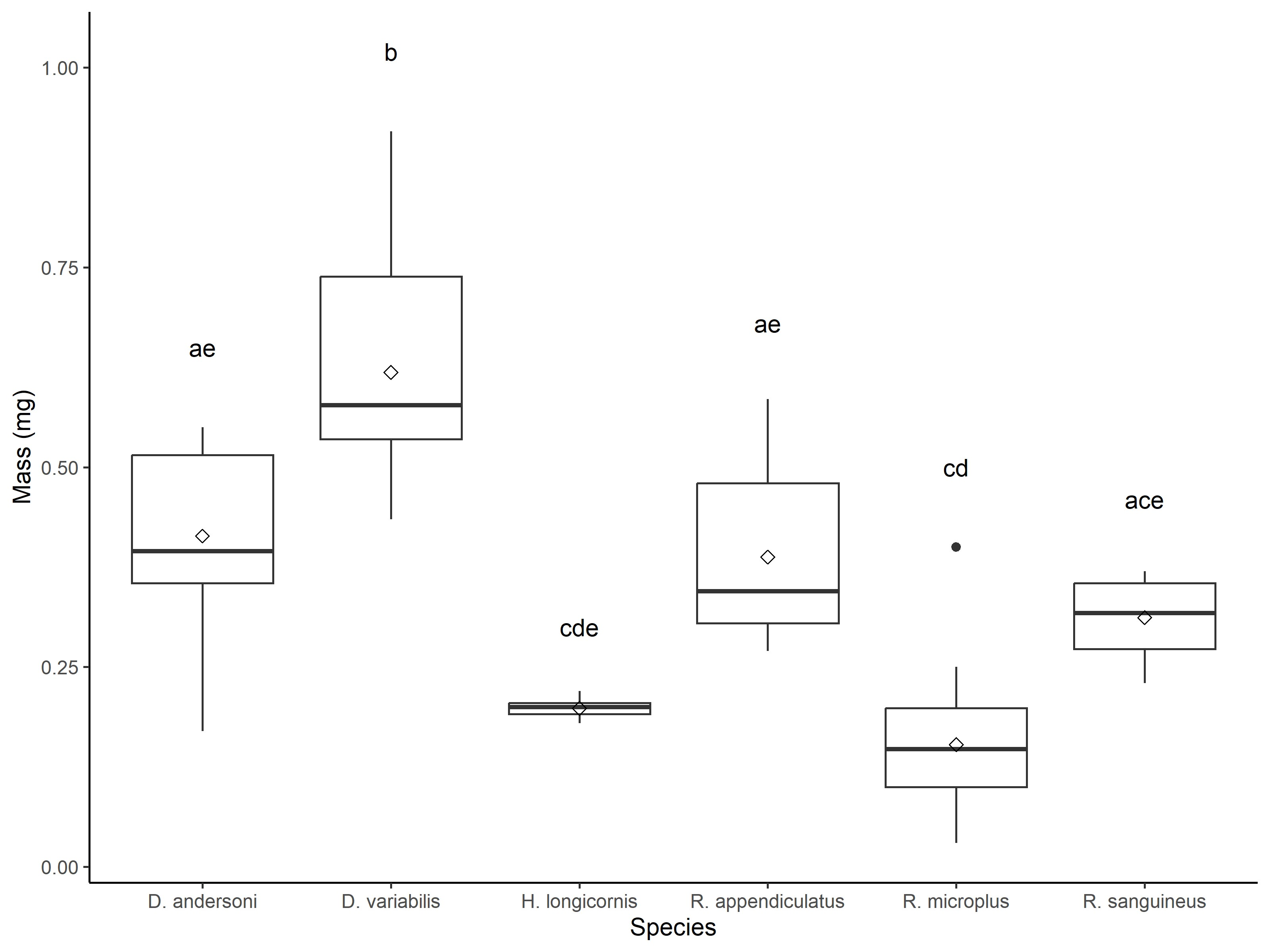


**Supplemental Figure 1.** Average wet body mass (mg) for each measured species. Sex was not significant across species in determining body mass (p > 0.05). Boxes represent 1^st^ to 3^rd^ quartile with the median indicated by the center line and the mean represented by the diamond. Extending lines represent 25^th^ to 75^th^ percentiles of the data. Points beyond are considered outliers. Boxes with different letters are significantly different from each other (p < 0.05).


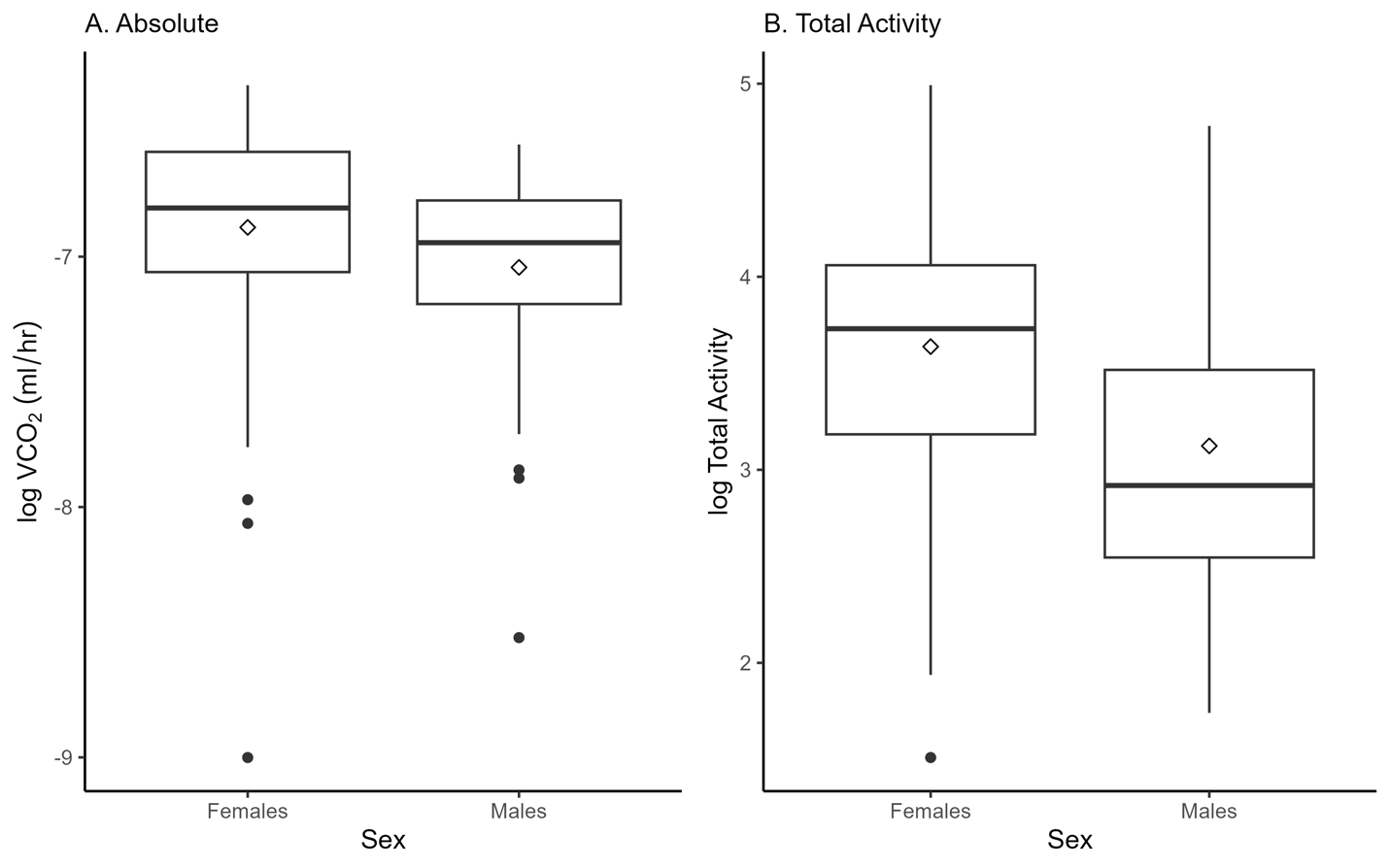


**Supplemental Figure 2.** Differences in total activity between female and male ticks. (A) Females had higher absolute VCO_2_ than males (t= 2.65, df = 170, p = 0.009). (B) Females were more active than males (t = 4.54, df = 139, p < 0.0001). Boxes represent 1^st^ to 3^rd^ quartile with the median indicated by the center line and the mean represented by the diamond. Extending lines represent 25^th^ to 75^th^ percentiles of the data. Points beyond are considered outliers.


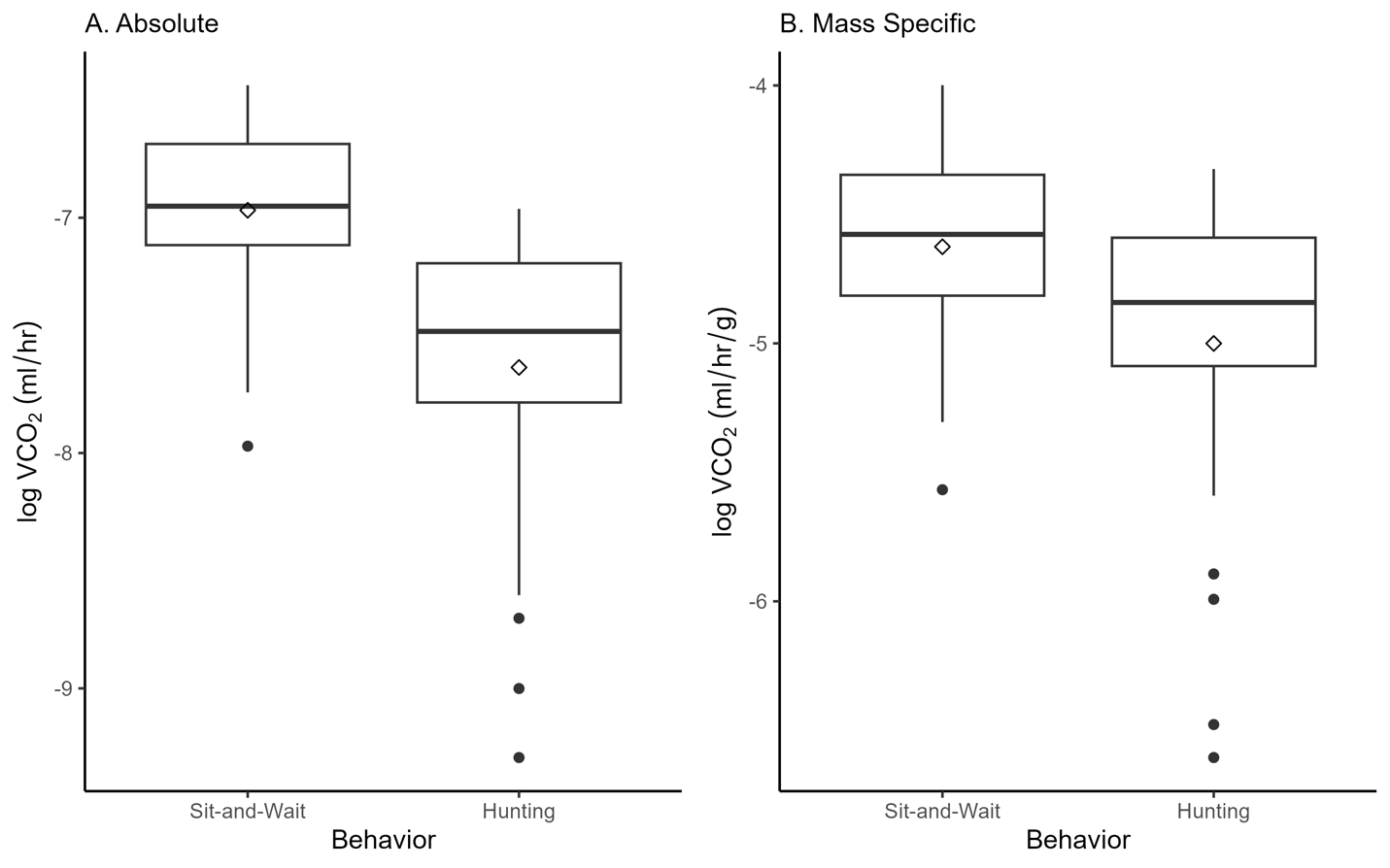


**Supplemental Figure 3.** Tick species were grouped based on published literature into either sit-and-wait or hunting questing behaviors. *Rhipicephalus microplus* was excluded from the analysis. (A) Absolute VCO_2_ was significantly different between the two behaviors (ANOVA; F_1,112_ = 50.4, p < 0.0001). (B) Mass-specific VCO_2_ was significantly different (ANOVA; F_1,112_ = 15.8, p = 0.0001). Means for each behavior is indicated by the diamonds.


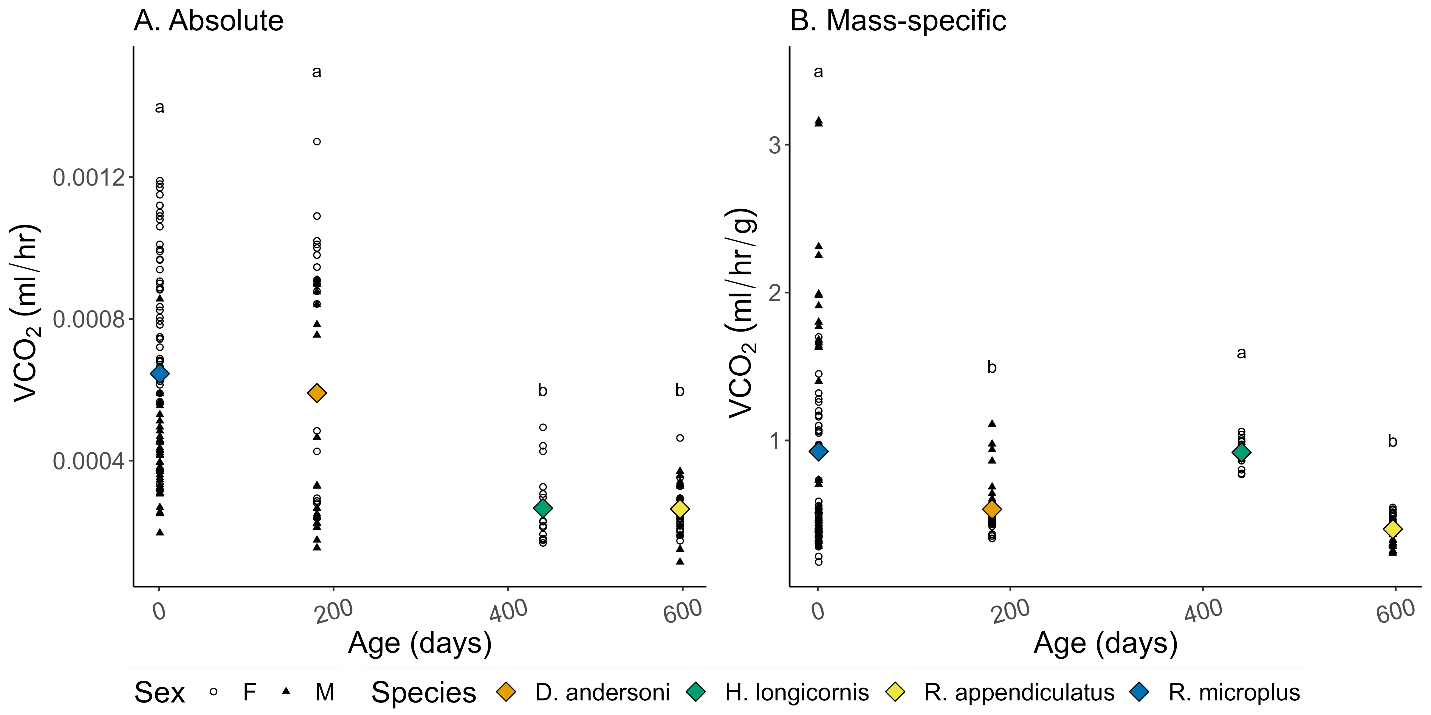


**Supplemental Figure 4.** Differences in VCO_2_ based on the age of ticks since last feeding. Only four species had known feeding dates, and the other two species were excluded. Means per species is indicated by the colored diamonds. Males are represented by black triangles, while females are open circles. (A) Absolute VCO_2_ was higher in species that had recently fed, *D. andersoni* and *R. microplus* (F_3,162_ = 22.1, p < 0.0001). (B) Mass-specific VCO_2_ differed between species; however, VCO_2_ did not increase or decrease with time from last feeding (F_3,162_ = 11.9, p < 0.0001). Points with different letters are significantly different from each other (p < 0.05).
