# Supplementary material for: Metabolic rate does not scale with body size or activity in some tick species
Source: Exp Appl Acarol. 2024 Sep 17;93(4):869–85. doi: 10.1007/s10493-024-00958-9 (PMC11534985; doi:10.1007/s10493-024-00958-9)
Supplement: Supplementary file 2 — Supplementary Material 2 [file 10493_2024_958_MOESM2_ESM.docx]

**Supplemental Figures**


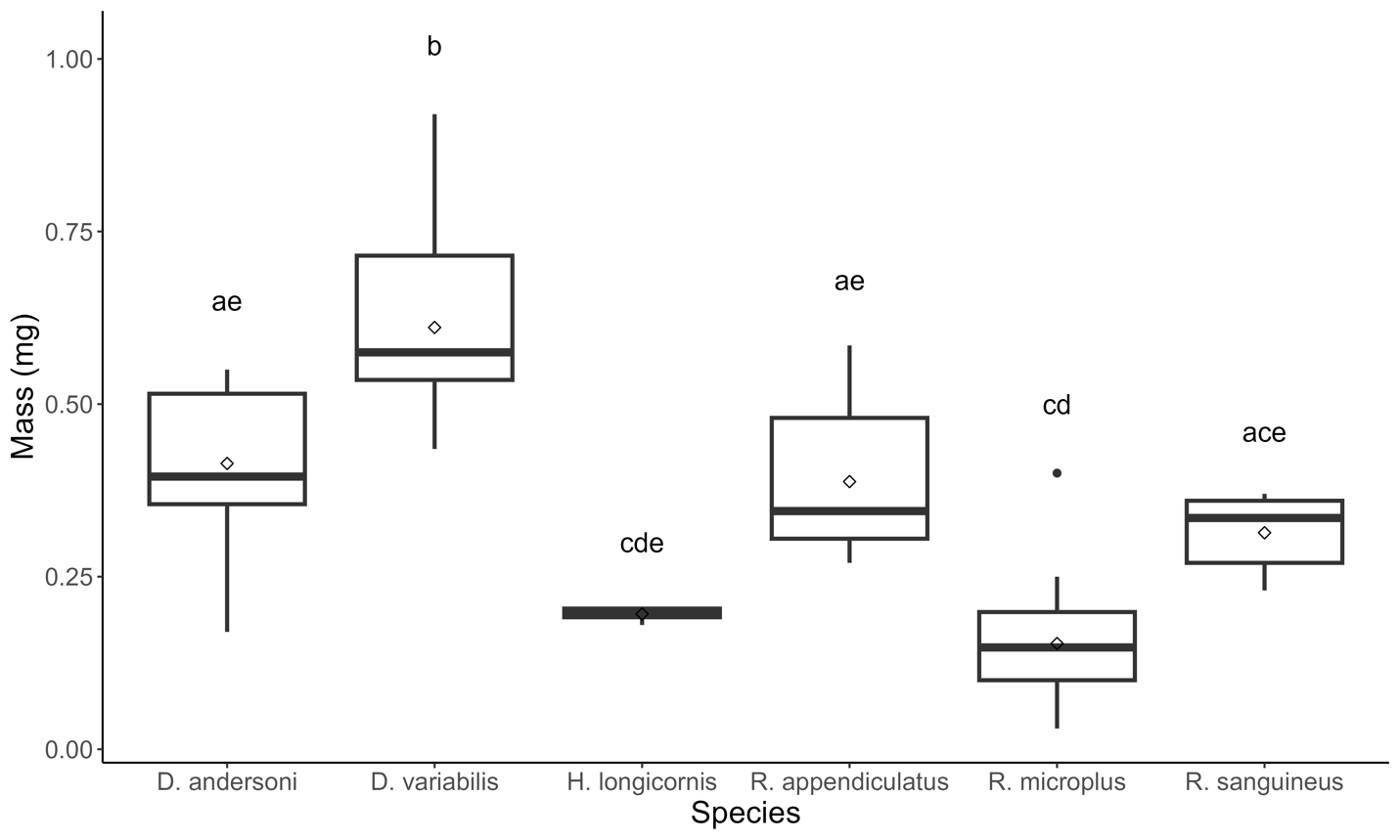


**Supplemental Figure 1.** Average wet body mass (mg) for each measured species. Sex was not significant across species in determining body mass (p > 0.05). Boxes represent 1^st^ to 3^rd^ quartile with the median indicated by the center line and the mean represented by the diamond. Extending lines represent 25^th^ to 75^th^ percentiles of the data. Points beyond are considered outliers. Boxes with different letters are significantly different from each other (p < 0.05).


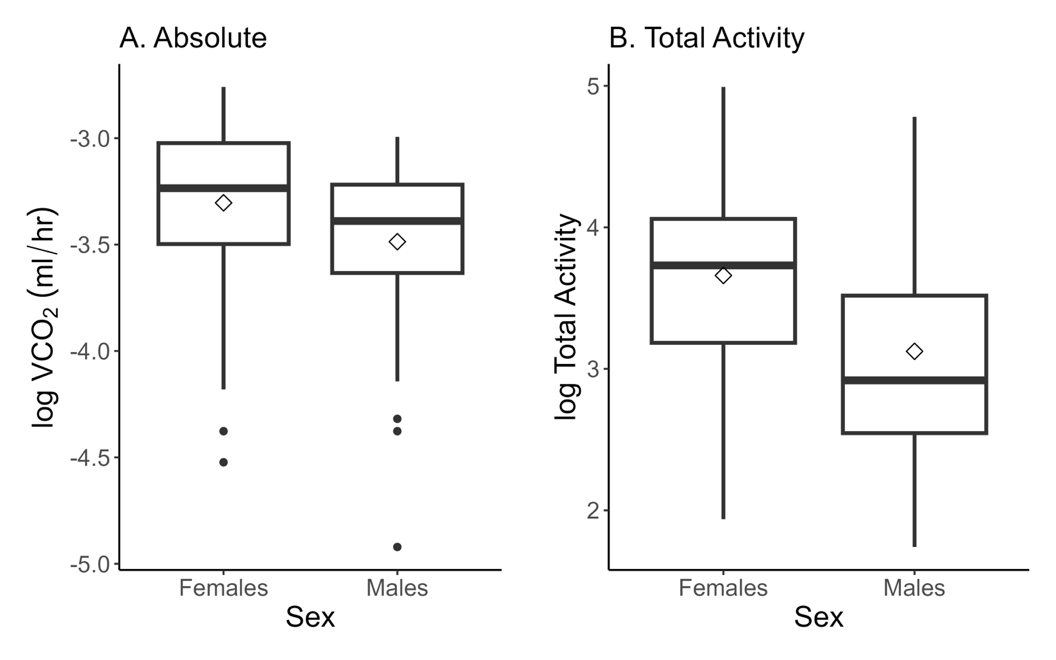


**Supplemental Figure 2.** Differences in total activity and absolute VCO_2_ between female and male ticks. (A) Females had higher absolute VCO_2_ than males (t= 3.25, df = 161, p = 0.001) when excluding the parthenogenic species *H. longicornis*. (B) Females were more active than males (t = 4.54, df = 139, p < 0.0001). Boxes represent 1^st^ to 3^rd^ quartile with the median indicated by the center line and the mean represented by the diamond. Extending lines represent 25^th^ to 75^th^ percentiles of the data. Points beyond are considered outliers.


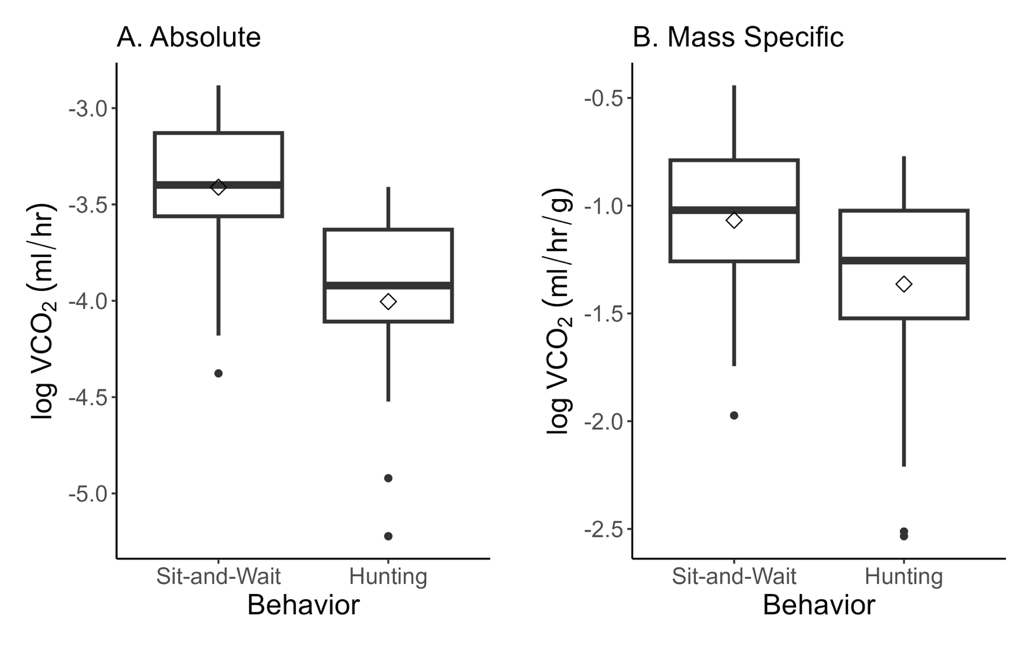


**Supplemental Figure 3.** Tick species were grouped based on published literature into either sit-and-wait or hunting questing behaviors. *Rhipicephalus microplus* was excluded from the analysis. (A) Absolute VCO_2_ was significantly different between the two behaviors (t-test; t = 5.44. df = 30, p < 0.0001). (B) Mass-specific VCO_2_ was significantly different (t-test; t = 2.79, df = 30.6, p = 0.009). Means for each behavior is indicated by the diamonds.

**Supplemental Table 1.** Global models for absolute and mass-specific VCO_2_ that was corrected for time since feeding. Models include replicate as a random factor to account for repeated measurements of individual ticks.

|  | ***Model Term*** | ***Estimate*** | ***Std error*** | ***T value*** | ***P value*** |
| --- | --- | --- | --- | --- | --- |
| ***Absolute VCO_2_*** | Intercept | -0.006 | 0.093 | -0.064 | 0.949 |
|  | Species (*H. longicornis*) | -0.529 | 0.120 | -4.40 | < 0.0001 |
|  | Species (*R. appendiculatus*) | 0.224 | 0.102 | 2.20 | 0.030 |
|  | Species (*R. microplus*) | 0.028 | 0.083 | 0.340 | 0.735 |
| ***Mass-Specific VCO_2_*** | Intercept | -0.293 | 0.103 | -2.85 | 0.005 |
|  | Species (*H. longicornis*) | -0.037 | 0.114 | -0.325 | 0.746 |
|  | Species (*R. appendiculatus*) | 0.519 | 0.100 | 5.40 | < 0.0001 |
|  | Species (*R. microplus*) | 0.384 | 0.078 | 4.91 | < 0.0001 |
